# Out of Pocket Expenditure for Sick Newborn Care in Referral Hospitals of Nepal

**DOI:** 10.1007/s10995-020-02881-y

**Published:** 2020-01-24

**Authors:** Avinash K. Sunny, Rejina Gurung, Abhishek Gurung, Omkar Basnet, Ashish KC

**Affiliations:** 1 Golden Community, Lalitpur, Nepal; 2 International Maternal and Child Health, Department of Women’s and Children’s Health, University Hospital, 751 85 Uppsala, Sweden

**Keywords:** Catastrophic health expenditure, Neonatal care, Nepal, Out-of-pocket expenditure

## Abstract

**Background:**

Almost all preventable neonatal deaths take place in low- and middle-income countries and affect the poorest who have the least access to high quality health services. Cost of health care is one of the factors preventing access to quality health services and universal health coverage. In Nepal, the majority of expenses related to newborn care are borne by the caregiver, regardless of socioeconomic status. We conducted a study to assess the out of pocket expenditure (OOPE) for sick newborn care in hospitals in Nepal.

**Methods:**

This cross-sectional study of hospital care for newborns was conducted in 11 hospitals in Nepal and explored OOPE incurred by caregivers for sick newborn care. Data were collected from the caregivers of the sick newborn on the topics of cost of travel, accommodation, treatment (drugs, diagnosis) and documented on a sick newborn case record form.

**Results:**

Data were collected from 814 caregivers. Cost of caregivers’ stay accounted for more than 40% of the OOPE for sick newborn care, followed by cost of travel, and the baby’s stay and treatment. The overall OOPE ranged from 13.6 to 226.1 US dollars (USD). The median OOPE was highest for preterm complications ($33.2 USD; CI 14.0–226.1), followed by hyperbilirubinemia ($31.9 USD; CI 14.0–60.7), respiratory distress syndrome ($26.9 USD; 15.3–121.5), neonatal sepsis ($ 25.8 USD; CI 13.6–139.8) and hypoxic ischemic encephalopathy ($23.4 USD; CI 13.6–97.7).

**Discussion for practice:**

In Nepal, OOPE for sick newborn care in hospitals varied by neonatal morbidity and duration of stay. The largest proportion of OOPE were for accommodation and travel. Affordable and accessible health care will substantially reduce the OOPE for sick newborn care in hospitals.

## Significance

The Sustainable Development Goal target to reduce neonatal mortality to less than 12 per thousand live births by 2030 will not be achieved unless there is access to high quality health services in low- and middle-income countries (LMICs). This paper highlights catastrophic out of pocket expenditures (OOPEs) related to newborn care. The expenses are not related to socioeconomic status and there is an urgent need to address the expenses borne by the caregivers for accommodation, travel and medication.

## Introduction

Globally, each year 19 million neonates have life-threatening conditions including intrapartum related brain injury, pathological jaundice, severe bacterial infections and preterm birth which require specialized care (Bhutani et al. 2013; Blencowe et al. 2013; Lawn et al. 2013; Lee et al. 2013; Seale et al. 2013). Of these, 2.9 million neonates die due to complications from preterm birth (34%), intrapartum-related conditions (25%), and infections (22%) (Lawn et al. 2014). Almost all of these deaths take place in LMICs and most of them occur in families who are poor and have least access to services (Lawn et al. 2005). Such financial barriers are a major obstacle that prevents families from accessing care at health institutions, especially impacting babies who require specialized care (Nabyonga et al. 2005; Pannarunothai and Mills 1997). Access to high quality, equitable and affordable health services is essential to meet the target of Sustainable Development Goal to reduce neonatal mortality to less than 12 per thousand live births by 2030 (Liu et al. 2016).

Over the last 15 years in Nepal, seeking health care for sick children has increased threefold, with more than 75% of sick children coming into contact with health institutions in 2014 (Nepal. Ministry of Health & Population, New Era, ICF Macro, & USAID 2011; Nepal. Ministry of Health & Population & UNICEF 2015). The expense of treatment of sick children in health facilities is taken up by families with no financial scheme from the government to reduce the OOPE (Nepal. Ministry of Health & Population & UNICEF 2015). As more than 55% of women give birth at a health institution, there has been increase in the need for special care of neonates with life threatening conditions. However, health facilities providing secondary and tertiary level neonatal care services for the management of sick newborns are not publicly funded. To further progress in reducing neonatal mortality so as to reach the target set by WHO Every Newborn Action Plan of 10 or less newborn deaths per 1000 live births by 2035, financial barriers for sick newborns needs to be removed coupled with improvement in quality of care (UNICEF 2014).

In Nepal, based on the readiness of health facilities, there are three levels of care (Nepal. Ministry of Health & Population 2010). Basic neonatal care in primary health centres where women come for delivery; secondary level care where sick newborns access newborn care units for management of neonatal sepsis, hyperbilirubinemia, preterm birth complication and hypoxic ischemic encephalopathy; and tertiary level care, where neonatal intensive care units exist to provide secondary level care and ventilator care.

Out-of-pocket expenditure (OOPE), defined as a fee made by an individual for a consultation with a health professional, an investigation or procedure, medicines, supplies and laboratory tests—accounts for a substantial amount of health care spending in many LMICs, particularly in Asia (World Health Organization 2010). In Bangladesh, India, China, and Vietnam, over 60% of health care costs are paid by OOPE (Van Doorslaer et al. 2006). Households in LMICs can experience extreme financial hardship and be pushed into poverty as a result of large and unexpected payments (Garg and Karan 2008; Saito et al. 2014; Van Doorslaer et al. 2006).

Nepal has been classified by the United Nations as one of the least developed countries, estimating a quarter of the population living below the poverty line (USD 1.25), with an annual per capita earning of USD 225 in 2013 (Malik 2013). In Nepal, about 6% of total household expenditure is spent on health care expenses. For those living below the poverty line, health care expenses account for more than 15% of total household expenditure, causing the family catastrophic health expenditures (Saito et al. 2014). In Nepal, such OOPE have been estimated to account for three-quarters or more of total expenditure on health (Van Doorslaer et al. 2007).

Currently, the health system of Nepal is funded through a mix of OOPE and free health care. The new National Health Policy (2014) confirms the Government of Nepal’s commitment to providing free universal health coverage and introducing a social security package with insurance schemes (Nepal. Ministry of Health and Population 2014). More than half of newborn babies have morbidity and the expenses incurred on neonatal illnesses are often higher than the family income (Srivastava et al. 2009). Babies born with low birth weight also incur higher medical expenses (Child Trends Data Bank 2012). There is some literatures exploring OOPE for delivery services in Nepal (Gartoulla et al. 2010, 2012). However, there is a lack of evidence on the out of pocket payments for neonatal intensive care services. This study was conducted to assess the OOPE for sick newborn care in referral hospitals (secondary level) in Nepal.

## Materials and Methods

This cross-sectional study was carried out in 11 hospitals among the total of 22 hospitals in the mid and far western region of Nepal between March and April 2015. Using each hospital as a single sampling unit, 50% of the hospitals in the region participated in the study. These hospitals were selected through simple random sampling. All sick babies who were admitted to the hospital during the study period were included in the study. Eight-hundred-fourteen caregivers were interviewed during the study period.

All the hospitals were government owned and secondary level health institutions that provided referral obstetric and neonatal services (Table 1). WHO standard protocol for essential newborn care and skilled birth attendance for newborn and maternal care were used in these hospitals (Nepal. Ministry of Health & Population 2007; World Health Organization & UNICEF 2017). These hospitals have a clinical protocol set for the initial assessment of newborns admitted for treatment which incorporates review of birth history including birth weight and birth complications during and after delivery. A clinical examination is also done to assess gestational age and newborn status.

**Table 1:** Site of assessment, annual delivery, stillbirth rate and neonatal mortality rate

Districts	Hospitals	Annual number of deliveries	Still birth rate (‘1000)	Neonatal mortality rate (‘1000)	Annual sick newborn admission
Kailali	Seti Zonal Hospital	5039	13.1	7	303
Kanchanpur	Mahakali Zonal Hospital	1819	19.2	9	110
Baitadi	Baitadi District Hospital	407	29.4	16	24
Bajhang	Bajhang District Hospital	2334	20.9	14	140
Achham	Achham District Hospital	299	83.6	43	18
Bajura	Bajura District Hospital	201	4.9	3	24
Doti	Doti District Hospital	398	23.0	12	27
Dadeldhura	Far-Western Sub-Regional Hospital	887	29.3	14	59
Banke	Bheri Zonal Hospital	4403	41.1	21	264
Surkhet	Mid-Western Regional Hospital	2862	25.1	15	171
Dang	Rapti Sub-Regional Hospital	3367	18.7	11	220

### Data Collection

Data were collected by a study team led by a research manager. There were two research officers placed in each hospital for data collection. Any sick baby admitted to the hospitals for treatment was eligible for inclusion. Information on clinical assessment, diagnosis and treatment along with gestational age, birth weight and sex of the baby was retrieved from clinical record forms. The study team then interviewed each caregiver (mother or other accompanying family member) of the sick baby at the time of discharge using a structured questionnaire to obtain social, demographic and household information (Central Bureau of Statistics 2011). Information on cost of travel, cost of accommodation of the caregiver and sick baby during the hospital stay, and expenditure made on drugs and diagnostics for the sick baby in the hospital were also obtained from the questionnaire. The structured interview was pretested for reliability among the 30 caregivers with a correlation coefficient of 0.70 on Cron Bach’s Alpha, suggesting good consistency and the validity of the tool was assessed with newborn experts. Based on the consistency and validity of the tool for use, the structured interview was used to collect data in this study.

### Variables

#### Cost of Newborn Care

This included cost of travel from home, cost of investigation and treatment, cost of supportive management, cost of accommodation for baby and the caregiver.

#### Maternal Age

Mothers were classified into age group of ≤ 20; 21–25; 26–30; 31–35 and ≥ 36 years.

#### Neonatal Morbidity

Sick babies were classified having any of the following morbidity:

- Preterm complication: any complication due to preterm birth i.e. Gestational age of the baby less than 37 completed weeks.
- Neonatal sepsis: clinical signs of severe bacterial infection, with a blood culture positive for a pathogenic organism.
- Hypoxic ischemic encephalopathy (HIE): syndrome of abnormal neurological behaviour in the neonate, which is frequently associated with multi-system dysfunction and follows severe injury before or during delivery.
- Respiratory distress syndrome (RDS): neonate with signs of respiratory distress-cyanosis, tachypnoea (> 60/min, shallow, rapid), grunting (delayed expiration maintains FRC), retraction (Subcostal, sub-sternal, intercostal), flaring (50% airway resist in nose& pharynx).
- Hyperbilirubinemia: babies with total Serum Bilirubin (TSB) increasing by > 5 mg/dl/day or 0.5 mg/dl/h, TSB > 15 mg/dl, conjugated serum bilirubin > 2 mg/dl.
- Meconium aspiration syndrome (MAS): breathing problems that a newborn baby may have when there are no other causes, and the baby has passed meconium (stool) into the amniotic fluid during labour or delivery.
- Major congenital malformation: a major physical defect seen in baby at birth which involves different part of body such as neuro-muscular, cardiac, lungs and intestinal tract.

#### Gestational Age of the Newborn

Gestational age measurement was based on the mother’s last menstrual period.

#### Birth Weight

Weight of the baby was measured within an hour of delivery using an analog pan scale.

#### Caste/Ethnicity

The caste/ethinicity of the baby and the mother was classified according to the social hierarchical system of caste which exists in Nepal (World Bank & DFID 2006). The ethnicity was categorized into six groups, as Brahmin/Chettri from the hill or terai region; relatively advantaged Janjatis, like Newar, Gurung and Thakali; disadvantaged Janjatis; other caste groups from the terai region; Dalit from the hill or terai region; and Muslim.

#### Wealth Index

The wealth index is a measure of socioeconomic status, which has been used for comparing socioeconomic inequalities in various national representative health surveys (Demographic Health Surveys) (Howe et al. 2009; Rutstein and Johnson 2004). Data on ownership of durable assets such as bicycle, car, radio, television, refrigerator; housing characteristics such as number of rooms, materials used for dwelling floor and roof, toilet facilities; and access to services such as electricity supply and drinking water source were assessed for scoring and constructing assest index. Caregivers were classified on the basis of value of these indices into population quintiles using cut-off values and ranked in order from poorest, poor, middle income, wealthy and wealthiest (Filmer and Pritchett 2001).

### Data Management

The interview forms were reviewed by the database manager to check for completeness and indexed for data safety and security. The data were entered and cleaned using the Census and Survey Processing System (CS Pro) software and exported to the IBM Statistical Package for Social Sciences (SPSS) software for data analysis.

### Data Analysis

Maternal age was analysed both as a continuous variable and as a categorical variable, grouping women into five groups as follows: < 20, 21–25, 26–30, 31–35 and 36 years of age and higher. Birth weight was categorized into grams less than 1500, 1500–2000, 2001–2500, 2501–3000, 3001–3500, 3501–4000, 4001 and more. The expenses in Nepalese rupees were converted to US dollars with an exchange rate of 107 Nepalese rupees (Nepal Rastra Bank 2015).

Descriptive statistics were presented with mean, standard deviation (SD), median, interquartile range (IQR), frequency and percentage. For comparing the average OOPE across the categories of various variables in this study, the Kruskal–Wallis test was used.

## Results

A total of 814 caregivers were interviewed during the study period. Mothers were generally young with an average of 23 years and the majority were from Brahmin and Chhetri castes (n = 355, 43.6%). The average birth weight of babies in this sample was 2689.0 g (SD 537.4). More than half of these babies (n = 433, 53.2%) were from two Zonal hospitals which are referral hospitals that have greater bed capacity and manage more complicated cases. Over half of these babies (n = 426, 52.3%) were admitted in intensive care due to neonatal sepsis (Table 2).

**Table 2:** Social and demographic profile of mothers and babies (n = 814)

Variables	N (%)
Maternal age (years)	
Mean ± SD	23.3 ± 4.1
≤ 20	235 (28.9)
21–25	378 (46.4)
26–30	156 (19.2)
31–35	37 (4.5)
≥ 36	8 (1.0)
Birth weight (g) (n = 806)	
≤ 1500	57 (7.0)
1501–2000	23 (2.8)
2001–2500	253 (31.1)
2501–3000	302 (37.1)
3001–3500	143 (17.6)
3501–4000	27 (3.3)
≥ 4001	1 (0.1)
Sex of baby	
Male	384 (47.2)
Female	430 (52.8)
Caste/ethnicity	
Brahmin/Chhetri (Hill and Terai)	355 (43.6)
Relatively advantaged Janajtis (Newar, Gurung, Thakali)	18 (2.2)
Disadvantaged Janjatis	108 (13.3)
Other Terai caste groups	159 (19.5)
Dalit (Hill and Terai)	156 (19.2)
Muslim	18 (2.2)
Wealth quintile	163 (20.0)
Wealthiest	163 (20.0)
Wealthy	163 (20.0)
Middle income	163 (20.0)
Poor	163 (20.0)
Poorest	162 (19.9)
Hospital	
Bheri Zonal Hospital	249 (30.6)
Seti Zonal Hospital	184 (22.6)
Rapti Sub-Regional Hospital	140 (17.2)
Mahakali Zonal Hospital	98 (12.0)
Bajhang District Hospital	38 (4.7)
Doti District Hospital	31 (3.8)
Surkhet Mid-Western Regional Hospital	21 (2.6)
Achham District Hospital	18 (2.2)
Dadeldhura Far-Western Sub-Regional Hospital	14 (1.7)
Baitadi District Hospital	13 (1.6)
Bajura District Hospital	8 (1.0)
Duration of stay (days)	
Median (IQR)	3.00 (1–27)
1–3	564 (69.3)
4–6	191 (23.5)
7–9	38 (4.7)
10 and more	21 (2.6)
Neonatal morbidity	
Preterm complication	53 (6.5)
Neonatal sepsis	426 (52.3)
Hypoxic ischemic encephalopathy	134 (16.5)
Respiratory distress syndrome	51 (6.3)
Hyperbilirubinemia	26 (3.2)
Meconium aspiration syndrome	118 (14.5)
Major congenital malformation	3 (0.4)
Other	3 (0.4)

**Fig. 1:**
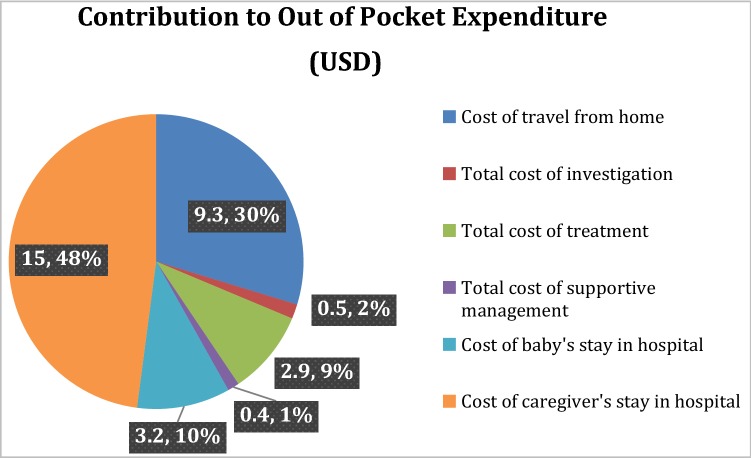
Contribution to out of pocket expenditure (USD)

The OOPE reported ranged from USD 13.6 to 226.1 with a mean of USD 31.3. The largest contributor to OOPE was “cost of caregivers’ stay in hospital”, accounting almost half of the reported mean total OOPE with USD 15 (48%). The next most common reason reported was for “cost of travel from home” with USD 9.3 (30%), followed by cost of baby’s stay with USD 3.2 (10%) and cost of treatment with USD 2.9 (9%) (Fig. 1).

OOPE was compared among various groups of ethnicities, wealth quintiles, maternal age, neonatal morbidity, birth weight, hospitals and duration of stay. It was highest among the disadvantaged Janjati with a median (IQR) of USD 35.8 (13.6–179.9). It was lowest among other terai caste groups with a median (IQR) of USD 23.4 (13.6–107.5) and Muslim with a median (IQR) of USD 23.5 (14.0–82.1) (p < 0.001).

Across wealth quintiles, OOPE was highest among poor and poorest with a median (IQR) of USD 25.7 (13.9–133.8) and USD 25.1 (13.6–107.5) respectively (p = 0.36). The OOPE for sick newborn varied across different age group of mothers and it was highest among mothers of 21–25 years with a median (IQR) of USD 25.5 (13.6–143.2) followed by mothers ≤ 20 years with a median (IQR) of USD 23.8 (13.6–226.1) (p = 0.25).

Caregivers’ whose babies suffered from preterm complications reported spending the highest out-of-pocket with a median (IQR) of USD 33.2 (14.0–226.1) followed by hyperbilirubinemia with a median (IQR) of USD 31.9 (14.0–60.7), respiratory distress syndrome with a median (IQR) of USD 26.9 (15.3–121.5), neonatal sepsis with USD a median (IQR) of 25.8 (13.6–139.8) and hypoxic ischemic encephalopathy with a median (IQR) of USD 23.4 (13.6–97.7) (p < 0.001). It was highest with a median (IQR) of USD 32.4 (14.0–226.1) among the babies with birthweight ≤ 1500 (p = 0.12). By hospitals, OOPE varied with a median (IQR) from USD 18.7 (14.0–51.4) to USD 33.0 (14.0–79.8) (p < 0.001) and it was seen to increase with duration of stay with a median (IQR) of USD 21.5 (13.6–62.9) for 1–3 days to USD 105.2 (56.1–226.1) for 10 days and more (p < 0.001) (Table 3).

**Table 3:** Out of pocket expenditure by ethnicity, wealth quintile, maternal age, neonatal morbidity, birth weight, hospital and duration of stay

Out of pocket expenditure (USD)	Mean ± SD	Median (IQR)	p value
Caste/ethnicity			
Brahmin/Chhetri (Hill and Terai)	29.9 ± 20.4	23.4 (13.6–226.1)	< 0.001
Relatively advantaged Janjatis (Newar, Gurung, Thakali)	31.6 ± 26.4	23.4 (13.9–121.5)	
Disadvantaged Janjatis	41.0 ± 27.3	35.8 (13.6–179.9)	
Other Terai Caste groups	29.0 ± 16.7	23.4 (13.6–107.5)	
Dalit (Hill and Terai)	30.5 ± 17.8	24.3 (14.0–105.2)	
Muslim	30.1 ± 18.3	23.5 (14.0–82.1)	
Wealth quintile			0.36
Wealthiest	33.1 ± 23.2	23.5 (13.6–179.9)	
Wealthy	32.9 ± 25.4	23.8 (14.0–226.1)	
Middle income	29.5 ± 18.7	23.4 (13.6–123.3)	
Poor	31.4 ± 19.4	25.7 (13.9–133.8)	
Poorest	29.9 ± 15.4	25.1 (13.6–107.5)	
Maternal age (years)			0.25
≤ 20	32.7 ± 25.2	23.8 (13.6 –226.1)	
21–25	31.1 ± 18.5	25.5 (13.6 –143.2)	
26–30	31.0 ± 19.2	23.4 (14.0 –133.8)	
31–35	27.9 ± 17.4	20.9 (14.0 –71.7)	
≥ 36	25.4 ± 16.3	18.0 (14.0 –54.2)	
Neonatal morbidity			< 0.001
Preterm complication	52.8 ± 46.4	33.2 (14.0–226.1)	
Neonatal sepsis	30.1 ± 15.6	25.8 (13.6–139.8)	
Hypoxic ischemic encephalopathy	31.2 ± 18,2	23.4 (13.6–97.7)	
Respiratory distress syndrome	35.9 ± 19.5	26.9 (15.3–121.5)	
Hyperbilirubinemia	31.2 ± 14.6	31.9 (14.0–60.7)	
Meconium aspiration syndrome	24.7 ± 16.8	19.6 (13.6–143.2)	
Major congenital malformation	26.2 ± 10.6	21.5 (18.7–38.4)	
Other	21.3 ± 10.9	16.0 (14.0–33.8)	
Birth weight (g)			0.12
≤ 1500	50.3 ± 45.7	32.4 (14.0–226.1)	
1501–2000	27.7 ± 11.5	28.0 (14.0–56.5)	
2001–2500	31.3 ± 19.1	23.4 (13.6–143.2)	
2501–3000	30.0 ± 16.7	24.1 (13.6–139.8)	
3001–3500	28.7 ± 13.4	23.4 (14.0–79.4)	
3501–4000	25.4 ± 10.9	20.9 (14.0–55.4)	
≥ 4001*	N/A	N/A	
Hospital			< 0.001
Bheri Zonal Hospital	41.3 ± 29.8	31.7 (13.6–226.1)	
Seti Zonal Hospital	20.0 ± 7.0	18.7 (14.0–51.4)	
Rapti Sub-Regional Hospital	34.2 ± 13.6	33.0 (14.0–79.8)	
Mahakali Zonal Hospital	28.5 ± 11.6	23.4 (14.0–61.7)	
Bajhang District Hospital	23.2 ± 9.1	23.4 (14.0–56.1)	
Doti District Hospital	28.7 ± 21.5	23.4 (14.0–121.5)	
Surkhet Mid-Western Regional Hospital	32.0 ± 16.1	28.0 (14.0–77.7)	
Achham District Hospital	31.9 ± 14.5	31.1 (14.0–54.9)	
Dadeldhura Far-Western Sub-Regional Hospital	24.2 ± 7.3	23.4 (18.7–41.4)	
Baitadi District Hospital	28.3 ± 7.3	31.3 (14.0–36.3)	
Bajura District Hospital	29.8 ± 6.6	30.4 (18.7–37.4)	
Duration of stay (days)			< 0.001
1–3	22.3 ± 7.8	21.5 (13.6–62.9)	
4–6	42.9 ± 11.9	41.4 (23.4–86.8)	
7–9	61.4 ± 15.6	56.2 (42.1–107.5)	
10 and more	114.0 ± 3.7	105.2 (56.1–226.1)	

*N = 1, therefore excluded from analysis

## Discussion

This study found that the OOPE for the treatment of sick babies in referral hospitals varied significantly with duration of stay and by neonatal morbidity. This is potentially explainable as the type of sickness determines the duration of stay in the hospital as well as the diagnostics and treatment used. The OOPE also varied significantly with hospitals and by ethnicity. Hospitals had different rates of admission for sick newborn resulting in varied OOPE. Similarly, there was varying proportion of sick babies of different ethnicity. The type of illness also varied according to ethnicity which suggests a significant difference in OOPE. To our knowledge, this is the one of the study conducted in Nepal on OOPE for sick newborn care in referral hospitals. There have been studies exploring OOPE for sick newborn in referral hospitals in South Asia. A study conducted in Bangladesh based on different cross-sectional surveys between 2009 to 2012 found the median OOPE for seeking care for a sick under-five child between USD 0.82–1.22 per child/visit (Tahsina et al. 2017). Medicine contributed the major portion of overall OOPE and higher overall OOPE for care seeking was associated with a priority illness which is consistent to findings in this study. A study conducted in India on OOPE to deliver public health facilities showed that an overall median OOPE was USD 11.48 (Issac et al. 2016). Similar to this study, a significant predictor for high OOPE was caste.

We found that the OOPE for the treatment of sick babies in referral hospitals varied significantly depending on duration of stay and by neonatal morbidity. This may be explained by the type of sickness which determines the duration of stay in the hospital as well the intensity of diagnostics and treatment used.

The OOPE also varied significantly by hospitals and ethnicity. Hospitals had different rates of admission for sick newborns resulting in varied OOPE. Similarly, there was varying proportion of sick babies by different ethnicity. The type of illness also varied according to ethnicity which suggests a significant difference in OOPE.

There are certain methodological limitations for the study. Firstly, our study includes only the public hospitals in mid-western and far-western region of Nepal, therefore, our results are not representative of private facilities. Secondly, the study did not capture the total OOPE that the family paid for the sick newborn i.e. the treatment cost taking place outside the hospital, which may have led to under-representation of actual OOPE. Thirdly, since the interview was taken from the family members about the payment made for the drugs, diagnostics, accommodation and travel and was not based on the actual receipt of the payment, there might have been recall bias (Lu et al. 2009). Lastly, since the sick babies were selected from March to April, there could have been selection bias due to seasonal variation in the occurrence of illness and the differences in transport expenses at various hospitals with different topography.

Despite these limitations, we have found major contributors to the OOPE for sick newborn care in secondary level hospitals. Cost of caregivers’ stay in hospital and cost of travel from home were the major contributors to OOPE. Most of the caregivers travelled far distances to the hospital via ambulance, bus or through hiring a private vehicle. Caregivers are not allowed to stay in hospital and thus they need to rent a room at a guest house nearby hospital. The expenses incurred in these indirect costs are higher compared to direct costs incurred in medical expenses of drugs and diagnostics. Studies have suggested the indirect cost of care seeking can exceed the direct costs to OOPE (Ensor and Cooper 2004; McIntyre et al. 2005). The findings from this study suggest the need for further research surrounding the cost of care for sick newborns in referral hospitals and the total OOPE incurred by families in caring for their newborns.

## Abbreviations

ANC: Antenatal care
LMICs: Low- and middle-income countries
OOPE: Out of pocket expenditure
SDG: Sustainable Development Goals
USD: US dollars
WHO: World Health Organization
